# Artificial intelligence, recessionary pressures and population health

**DOI:** 10.2471/BLT.24.291950

**Published:** 2025-01-02

**Authors:** Jo-An Occhipinti, Ante Prodan, William Hynes, John Buchanan, Roy Green, Sharan Burrow, Harris A Eyre, Adam Skinner, Ian B Hickie, Mark Heffernan, Yun Ju Christine Song, Goran Ujdur, Marcel Tanner

**Affiliations:** a Mental Wealth Initiative, Brain and Mind Centre, Faculty of Medicine and Health, University of Sydney, Level 4, Moore College CG2, 1 King Street, Newtown, NSW, 2042 Australia.; b School of Computer, Data and Mathematical Sciences, Western Sydney University, Sydney, Australia.; c World Bank, Paris, France.; d Business School, University of Sydney, Sydney, Australia.; e UTS Business School, University of Technology Sydney, Sydney, Australia.; f London School of Economics Grantham Institute, London, England.; g Baker Institute for Public Policy, Rice University, Houston, United States of America.; h Swiss Academies of Arts and Sciences, Bern, Switzerland.

## Abstract

Economic and labour policies have a considerable influence on health and well-being through direct financial impacts, and by shaping social and physical environments. Strong economies are important for public health investment and employment, yet the rapid rise of generative artificial intelligence (AI) has the potential to reshape economies, presenting challenges beyond mere temporary market disruption. Generative AI can perform non-routine cognitive tasks, previously unattainable though traditional automation, creating new efficiencies. While this technology offers opportunities for innovation and productivity, its labour-displacing potential raises serious concerns about economic stability and social equity, both of which are critical to health. Job displacement driven by generative AI could worsen income inequality, shrink middle-class opportunities and reduce consumer demand, triggering recessionary pressures. In this article, we propose the existence of an AI-capital-to-labour ratio threshold beyond which a self-reinforcing cycle of recessionary pressures may emerge, and which market forces alone cannot correct. Traditional responses to such pressures, like fiscal stimulus or monetary easing, may be ineffective in addressing structural disruptions to labour markets caused by generative AI. We call for a proactive global response to harness the benefits of generative AI while mitigating risks. This response should focus on reorienting economic systems towards collective well-being, as emphasized in the World Health Assembly resolution *Economics of health for all* and the United Nations' Global Digital Compact. Integrated strategies that combine fiscal policy, regulation and social policies are critical to ensuring generative AI advances societal health and equity while avoiding harm from excessive job displacement.

## Résumé

Les politiques en matière d’économie et d’emploi exercent une influence considérable sur la santé et le bien-être par des impacts financiers directs ainsi qu’une transformation des environnements sociaux et physiques. Des économies solides sont primordiales pour investir dans la santé publique et l’emploi, mais l’essor rapide de l’intelligence artificielle (IA) générative a le potentiel de remodeler ces économies, ce qui pose des défis dépassant la simple perturbation temporaire du marché. L’IA générative est capable d’effectuer des tâches cognitives non routinières qui étaient autrefois impossibles à réaliser par l’automatisation traditionnelle, générant ainsi de nouvelles efficacités. Si cette technologie offre des possibilités d’innovation et de productivité, son potentiel de suppression de la main-d’œuvre soulève de graves préoccupations en matière de stabilité économique et d’équité sociale, deux facteurs essentiels à la santé. La suppression d’emplois induite par l’IA générative est de nature à aggraver les inégalités de revenus, à réduire les opportunités pour la classe moyenne et à mettre en berne la demande des consommateurs, mettant en place les conditions propices à une récession.  Le présent article suggère l’existence d’un seuil de ratio IA / capital / main-d’œuvre au-delà duquel un cycle de conditions propices à une récession risque d’émerger, cycle qui se renforce de manière indépendante et que les tendances du marché ne peuvent à elles seules corriger. Les réponses traditionnelles à ces pressions, telles qu’un plan de relance budgétaire ou un assouplissement monétaire, risquent de s’avérer inefficaces pour remédier aux perturbations structurelles des marchés du travail induites par l’IA générative. Nous appelons donc à une réponse mondiale proactive afin d’exploiter les avantages de l’IA générative tout en en atténuant les risques. Cette réponse devrait se concentrer sur la réorientation des systèmes économiques en faveur du bien-être collectif, comme le soulignent la résolution de l’Assemblée mondiale de la Santé intitulée *Économie de la santé pour tous* et le Pacte numérique mondial des Nations unies. Des stratégies intégrées alliant politique fiscale, réglementation et politiques sociales sont essentielles pour garantir que la santé et l’équité des sociétés progressent grâce à l’IA générative tout en évitant les effets néfastes d’une suppression excessive d’emplois.

## Resumen

Las políticas económicas y laborales influyen de forma considerable en la salud y el bienestar a través de sus repercusiones financieras directas y de la configuración de los entornos sociales y físicos. Las economías sólidas son importantes para la inversión en salud pública y el empleo, pero el rápido auge de la inteligencia artificial (IA)generativa tiene el potencial de remodelar las economías, lo que plantea desafíos que van más allá de la mera perturbación temporal del mercado. La IA generativa puede realizar tareas cognitivas no rutinarias, que antes eran inalcanzables mediante la automatización tradicional, y crear nuevas eficiencias. Si bien esta tecnología ofrece oportunidades para la innovación y la productividad, su potencial de desplazar mano de obra plantea serias preocupaciones sobre la estabilidad económica y la equidad social, ambas fundamentales para la salud. El desplazamiento laboral impulsado por la IA generativa podría agravar la desigualdad de ingresos, reducir las oportunidades para la clase media y disminuir la demanda de los consumidores, lo que desencadenaría presiones recesivas. En este artículo, se propone la existencia de un umbral en la relación entre el capital en IA y la mano de obra, más allá del cual podría surgir un ciclo autoreforzado de presiones recesivas que las fuerzas del mercado por sí solas no podrían corregir. Las respuestas tradicionales a estas presiones, como el estímulo fiscal o la flexibilización monetaria, pueden resultar ineficaces para abordar las disrupciones estructurales en los mercados laborales provocadas por la IA generativa. Por lo tanto, se hace un llamado a una respuesta global proactiva para aprovechar los beneficios de la IA generativa mientras se mitigan los riesgos. Esta respuesta debería centrarse en reorientar los sistemas económicos hacia el bienestar colectivo, como se enfatiza en la resolución de la Asamblea Mundial de la Salud sobre la *Economía de la salud para todos* y en el Pacto Digital Global de las Naciones Unidas. Las estrategias integradas que combinan política fiscal, regulación y políticas sociales son fundamentales para garantizar que la IA generativa promueva la salud y la equidad en la sociedad, evitando al mismo tiempo los daños derivados de un desplazamiento laboral excesivo.

## ملخص

توثر السياسات الاقتصادية والعمالية بشكل كبير على الصحة والرفاهية من خلال التأثيرات المالية المباشرة، ومن خلال تشكيل البيئات الاجتماعية والمادية. إن النظم الاقتصادية القوية تمثل أهمية للاستثمار والتوظيف في الصحة العامة، ومع ذلك فإن الصعود السريع للذكاء الاصطناعي التوليدي (AI) لديه القدرة على إعادة تشكيل النظم الاقتصادية، مما يمثل تحديات تتجاوز مجرد الاضطراب المؤقت في السوق. يمكن للذكاء الاصطناعي التوليدي أداء مهام معرفية غير روتينية، كانت سابقًا غير قابلة للتحقيق من خلال الأتمتة التقليدية، مما يؤدي لخلق كفاءات جديدة. في حين توفر هذه التكنولوجيا فرصًا للابتكار والإنتاجية، فإن احتمال أن تؤدي لإزاحة العمالة تثير مخاوف جدية بشأن الاستقرار الاقتصادي والمساواة الاجتماعية، وكلاهما أمر بالغ الأهمية بالنسبة للصحة. يمكن أن تؤدي إزاحة الوظائف الناجمة عن الذكاء الاصطناعي التوليدي إلى تفاقم عدم المساواة في الدخل، وتقليص فرص الطبقة المتوسطة، والحد من طلبات الاستهلاك، مما يؤدي إلى ضغوط الركود. في هذه المقالة، نقترح وجود عتبة لنسبة لرأس المال بين الذكاء الاصطناعي إلى العمالة، والتي قد تنشأ بعدها دورة ذاتية التعزيز من ضغوط الركود، والتي لا تستطيع قوى السوق وحدها تصحيحها. إن الاستجابات التقليدية لمثل هذه الضغوط، مثل التحفيز المالي أو التيسير النقدي، قد تكون غير فعّالة في معالجة الاضطرابات الهيكلية في أسواق العمل الناجمة عن الذكاء الاصطناعي التوليدي. ولذلك، فنحن ندعو إلى استجابة عالمية استباقية للاستفادة من مزايا الذكاء الاصطناعي التوليدي، إلى جانب تخفيف المخاطر. وينبغي أن تركز هذه الاستجابة على إعادة توجيه الأنظمة الاقتصادية نحو الرفاهية الجماعية، كما أكد قرار جمعية الصحة العالمية اقتصاد الصحة للجميع ( *Economics of health for all) *، والميثاق الرقمي العالمي للأمم المتحدة. تشكل الاستراتيجيات المتكاملة التي تجمع بين السياسة المالية، والتنظيم، والسياسات الاجتماعية أهمية بالغة لضمان قيام الذكاء الاصطناعي التوليدي بتعزيز الصحة المجتمعية والمساواة، مع تجنب الضرر الناجم عن الإزاحة المفرطة للوظائف.

## 摘要

经济和劳工政策会直接影响财政并塑造社会和物质环境，从而对健康和福祉产生相当大的影响。强劲的经济是促进公共卫生投资和就业的重要保障，但生成式人工智能 (AI) 的迅速崛起有可能导致经济重塑，因此带来的挑战将不仅仅是暂时性的市场混乱。生成式 AI 可执行非常规认知任务（此为以往的传统自动化无法实现的功能），并将作业效率提高到了新的水平。虽然这项技术有利于促进创新和提高生产力，但其也有取代劳动力的发展趋势，该现象导致人们极其担心经济稳定性和社会公平性问题，而这又是对健康至关重要的两个问题。在生成式 AI 驱动下造成的工作岗位流失可能会加剧收入不平等现象，削减中产阶级的工作机会，并降低消费者需求，从而引发经济衰退的压力。在本文中，我们提出存在 AI 资本与劳动比率阈值，而如果超过该阈值，可能会出现经济衰退压力自我强化循环的情况，此时仅靠市场力量无法扭转局势。针对这种压力采取的传统应对措施（如财政刺激或货币宽松政策）可能无法有效解决生成式 AI 对劳动力市场造成的结构性破坏。因此，我们呼吁采取积极主动的全球应对措施，以确保在利用生成式 AI 的优势的同时降低风险。正如世界卫生大会决议“全民健康经济学”和联合国“全球数字契约”所强调的那样，这一应对措施应侧重于将经济体系重新定位为追求集体福祉。将财政政策与监管和社会政策相结合的综合战略对于确保生成式 AI 在促进社会健康和公平的同时避免因工作岗位过度流失而造成的伤害至关重要。

## Резюме

Экономическая политика и политика в сфере труда оказывают значительное влияние на здоровье и благополучие населения, оказывая прямое финансовое воздействие, а также формируя социальную и физическую среду. Крепкая экономика важна для инвестиций в здравоохранение и занятости населения, однако стремительное развитие генеративного искусственного интеллекта (ИИ) способно привести к перестройке экономики и создать проблемы, выходящие за рамки простого временного нарушения работы рынка. Генеративный ИИ может выполнять нестандартные когнитивные задачи, ранее недостижимые при традиционной автоматизации, создавая новые возможности для повышения эффективности. Хотя эта технология открывает возможности для развития инноваций и повышения производительности, ее потенциал замещения рабочей силы вызывает серьезные опасения в отношении экономической стабильности и социального равенства, которые имеют решающее значение для здоровья. Сокращение рабочих мест, вызванное генеративным ИИ, может усугубить неравенство доходов, уменьшить возможности среднего класса и снизить потребительский спрос, что приведет к экономическому спаду. В этой статье рассматривается вопрос о существовании порогового значения соотношения между капиталом и рабочей силой, за которым может возникнуть самоусиливающийся цикл экономического спада и который рыночные силы не в состоянии исправить. Традиционные меры реагирования на подобные процессы, такие как налогово-бюджетное стимулирование или смягчение денежно-кредитной политики, могут оказаться неэффективными для устранения структурных нарушений на рынках труда, вызванных генеративным ИИ. Поэтому призываем предпринять активные глобальные действия, чтобы использовать преимущества генеративного ИИ и одновременно снизить риски. Этот ответ должен быть направлен на переориентацию экономических систем на коллективное благосостояние, как подчеркивается в резолюции Всемирной ассамблеи здравоохранения *«Экономические аспекты концепции "Здоровье для всех"»* и Глобальном цифровом договоре ООН. Комплексные стратегии, сочетающие налогово-бюджетную политику, регулирование и социальную политику, имеют решающее значение для обеспечения стимулирования развития генеративного ИИ в интересах здорового и справедливого общества и предотвращения вреда от чрезмерного сокращения рабочих мест.

## Introduction

Economic structures and policies can have profound impacts on health and well-being, both directly through the psychological effects of financial hardship and indirectly by shaping our social and physical environments.^1^ For example, industrial relations reforms implemented since the 1980s to boost business flexibility, along with the deregulation of the financial sector to create a more competitive system, have increased job insecurity, intensified work pressures, raised household debt, reduced financial security and increased the vulnerability of individuals.^2^^,^^3^ These effects can influence health and well-being by disrupting social connections, leading to isolation.^4^ They may also escalate parental stress and family conflict,^5^ increasing the risk of substance misuse, family and domestic violence,^6^ and child abuse and neglect.^7^^,^^8^ Moreover, economic well-being is critical to the capacity of nations to invest in health and social care systems, public health initiatives and health innovations. Strong economies also provide stable, quality employment, serving as the foundation for societal cohesion, stability, resilience and prosperity.^9^ Exclusion from stable, high-quality work can cause poor psychological well-being, increasing the risk of deaths of despair.^10^^,^^11^ Guarding against economic downturns and the associated risks to employment is therefore a shared concern for economists, governments, business leaders, labour organizations, and a broad range of health sector actors, including public health professionals, health workers, researchers and policy experts.

The advent of generative artificial intelligence (AI), particularly large language models like GPT-3 (generative pre-trained transformer 3), launched by OpenAI (San Francisco, United States of America) in November 2022, has opened a new frontier in innovation, labour productivity and economic growth. However, this advancement has also sparked debate on whether generative AI-driven productivity growth represents an opportunity or a threat to social and economic well-being. In this paper, we highlight how generative AI has the potential to profoundly reshape labour market dynamics and paradoxically, if left to market dynamics, undermine the very economic growth and associated well-being it aims to achieve.

Many economists consider generative AI as comparable to previous technological advancements, such as the internet or computers, and emphasize this development as simply a continuity of the historical pattern of technology-driven progress. They argue that, like past innovations, generative AI is another form of capital that can augment human capabilities to enhance labour productivity, efficiency, economic growth and, ultimately, societal well-being. By citing historical precedents and analyses, they argue that technological advancements have typically resulted in the creation of new types of jobs. These advancements have also driven a gradual adjustment in the tasks performed by workers, the requisite skill levels, and the share of income earned, rather than an aggregate reduction in the amount of work available.^12^^,^^13^ Another fundamental assumption that underpins the optimistic view is that the new jobs created as a result of this technological disruption will require human input, and at a level to offset aggregate job loss. The World Economic Forum’s *Future of Jobs Report, 2023*, estimates that the largest drivers of job growth to offset job displacement will likely be in the areas of big data analytics; climate change and environmental management technologies; encryption and cybersecurity; education; agricultural professionals; e-commerce and trade.^14^ While this analysis has some merit, it reveals a fundamental misunderstanding of the capabilities of generative AI.

Generative AI is not simply automation; it is a form of intelligence rapidly evolving to acquire experiential learning, creative adaptability, strategic thinking and the ability to generate knowledge surpassing human capacities. Considerable improvements have been achieved with GPT-4 (released March 2023), showing an average 25% performance improvement across a broad range of task categories compared to GPT-3.5 released months earlier.^15^ Researchers have shown that GPT-4 is already capable of solving novel and complex tasks across various fields, by applying its transdisciplinary knowledge, without necessarily requiring specific prompting.^16^ On professional and academic exams, GPT-4 exhibits human-level performance scoring in the top 10% of test takers in a simulated version of the multistate bar examination.^16^^,^^17^ OpenAI is developing GPTs that are enhancing productivity not only in everyday tasks but also in professional assignments without the necessity of programming skills. Estimates from the Brookings Institution indicate that, within the next decade, around 60% of job tasks in the United States alone are at medium to high risk of being replaced by AI.^18^ While timelines for when the potential impacts of generative AI on the workforce and economy will be fully realized remain uncertain, substantial investments are being made (anticipated to surpass 300 billion United States dollars (US$) in 2026)^19^ to progress capability, reliability and implementation of generative AI, including the potential for self-advancement with minimal human input. Experts who understand the capability and trajectory of generative AI recognize that the current surge in AI-specialized jobs may ironically promote their own obsolescence.^20^ The pioneers of this technology are now openly acknowledging that generative AI is fundamentally a labour-replacing tool.^21^

To appreciate the likely impact of generative AI on the nature of work and the economy, it is helpful to first consider historic trends in four occupational categories: routine manual, non-routine manual, routine cognitive and non-routine cognitive.^12^ Routine cognitive jobs involve tasks that can be codified into programmable instructions and are more susceptible to automation, such as office, administrative and sales positions. In contrast, non-routine cognitive jobs require complex problem-solving, strategic thinking, synthesis of information and judgment, like managerial, professional and technical roles. Routine manual jobs include physical tasks that are repetitive and can be easily automated, such as assembly line work and data entry. Non-routine manual jobs require adaptability and physical dexterity, often involving tasks that are less predictable and harder to automate, such as those performed by electricians, plumbers and skilled tradespeople.

**Fig. 1:**
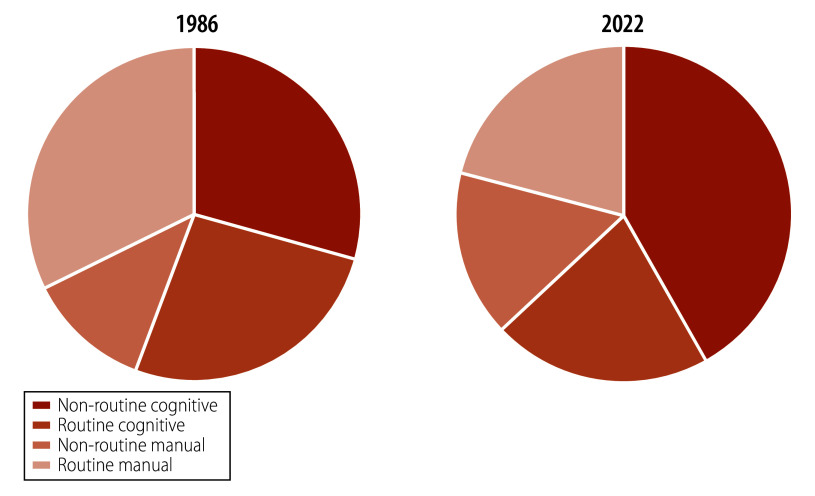
Share of employment by type of occupation, Australia, 1986 and 2022

Notes: Reprinted with permission from Borland & Coelli, 2023.^12^ Examples of non-routine cognitive jobs are data analysts, engineers, medical technicians, architects, journalists, writers, visual arts professionals, fashion designers, musicians, interior designers, advertising professionals, business, human resources and marketing professionals, accountants, auditors, financial investment advisers and managers, public relations professionals and legal professionals. Examples of routine cognitive jobs are clerical, administrative and sales roles, bookkeepers and loans officers. Examples of routine manual jobs are construction trade workers, machine operators and delivery drivers. Examples of non-routine manual jobs include protective services, food preparation, cleaning and personal care services.

Fig. 1 illustrates a typical example of the change in proportions of these jobs over time based on the Australian context, which is similar across other high-income countries. The share of employment consisting primarily of routine manual and routine cognitive tasks has declined with increasing automation, particularly with the advancements in information technology since the 1990s.^12^ However, offsetting this decline has been a 12-percentage-point increase in non-routine cognitive occupations between 1986 and 2022, demonstrating the creation of new higher-skilled, higher-paid jobs requiring human skills that could not be replaced by capital.^12^ The departure from this historical trend comes with the introduction of generative AI, which has the capacity to handle non-routine cognitive tasks. Unlike earlier automation technologies that were primarily suited for routine and repetitive manual and cognitive tasks, generative AI can analyse complex data, generate creative content and perform other non-routine functions at or above human-level performance.^16^

A broad range of professional and technical occupations have been identified as facing medium to high exposure to displacement by AI in the coming decade including accountancy, finance, banking, legal, human resources, teaching, data analysis, creative writing, marketing and communications, journalism and medical diagnostic roles.^22^ While generative AI is unlikely to eliminate labour in affected occupations, the need for human input will be substantially reduced, even for new non-routine cognitive jobs and sectors that may be created as a result of this technological disruption.^23^ As generative AI platforms become increasingly efficient, accurate and accepted, and costs continue to decrease, the economic incentive to create new jobs may diminish, with businesses potentially favouring generative AI solutions over human labour for new markets. While the deployment of generative AI may create new high-skilled occupations where human labour is preferred, such as those related to AI ethics, data privacy and algorithm oversight, opportunities in these occupations may not be sufficient to offset the scale of displacement of workers across cognitive occupations.^24^^,^^25^ With contraction of labour demand, increases in unemployment, underemployment, non-participation in the labour market and displacement to lower paid manual jobs, such as protective services, food, cleaning and care services, should be expected, with serious adverse consequences for population health, especially mental health due to an increase in financial hardship.

## Recessionary pressures

The amount of downward pressure on consumer demand will depend on the speed and scale of the shift in the AI-capital-to-labour ratio, that is, the substitution of human labour with AI-capital.^25^ Replacement of existing capital with AI-capital, or smaller increases in the aggregate ratio of AI-capital to labour across the economy are likely to simply augment labour productivity, lowering prices, increasing consumers’ real incomes, increasing demand for goods and services and creating employment opportunities. However, a threshold likely exists beyond which the substitution of labour with AI-capital (capital deepening) could trigger a self-reinforcing loop. The decreasing availability of higher-paid, high- and middle-skilled jobs will displace workers across a broad range of professions. This displacement may increase competition for lower-skilled jobs, leading to wage compression, reduced job quality, and the likely expansion of an insecure and exploitative gig economy. As a result, average household incomes will fall, reducing demand for goods and services, and exacerbating labour market contractions even in sectors not directly affected by generative AI. This downward pressure on labour demand could further destabilize the economy. Even among workers who retain high-skilled jobs, rising job insecurity will undermine consumer confidence, reducing discretionary spending. This reduction in spending will pose challenges for a broad range of businesses to maintain profitability, leading to decreased investment and greater volatility in financial markets. The labour share of income, which has already been in decline since the mid-1980s due to the impact of technology,^26^ may decrease further as owners of AI-capital claim a growing portion of national income. Increasing income inequality, job scarcity and associated migration of people seeking work, coupled with a contraction in brain capital and diminishing public trust in the economic system, are likely to intensify polarization and political unrest, posing additional challenges to social and economic stability.

Generative AI enables an aggregate decoupling of economic productivity from labour input, with its virtualized infrastructure capable of scaling in days instead of years. While this decoupling could enable rapid and continuous productivity growth, unconstrained increases in the AI-capital-to-labour ratio to sustain that growth would likely disrupt labour markets, undermine economic stability and harm individual and social well-being. Additionally, the potential for productivity growth is limited by the bounded demand for goods and services. Together, the displacement of labour and bounded demand create a paradox where increased productivity capabilities encounter a threshold beyond which demand falls.

## Additional considerations

### Social prosperity

In addition to contributing to recessionary pressures, more substantial increases in the AI-capital-to-labour ratio could pose a broad range of threats to social prosperity. For example, with middle- to high-income cognitive occupational categories constituting more than 60% of the total labour market in Australia, shrinking job opportunities in these categories would catalyse a middle-class contraction as well as an erosion of brain capital. Social mobility would decline, graduate opportunities and pathways for career development would become scarce, creating extreme competition in affected professions. These impacts could further exacerbate the youth mental health crisis. As seen recently among young people in China, extreme competition and declining economic opportunities can feed despondency, reduce engagement with higher education and increase a sense of hopelessness.^27^ Similar effects on young people, caused by diminished economic opportunities, were seen following austerity measures implemented after the 2008 global financial crisis.^28^^,^^29^ As generative AI becomes proficient in replicating a broader range of non-routine cognitive tasks, the value of degrees and certifications associated with certain cognitive skills and professions is likely to diminish, posing considerable risks to the traditional structures and functions of the tertiary education sector.

While generative AI presents challenges to social prosperity, it also offers benefits, particularly in sectors with skills shortages.^30^ For example, in the health-care sector, generative AI can complement traditional AI used for routine pathology screenings by performing complex diagnostic analyses and adapting to emerging medical data trends, thereby further enhancing health-care systems’ capacity to deliver timely and personalized care. This capability allows for a more effective allocation of human resources, enabling health workers to focus on patient care and decision-making. By addressing workforce shortages in given sectors, AI can support social well-being and complement human efforts to improve the quality and accessibility of essential services.

The risks associated with this technological transition lie in the scale of potential displacement across many sectors and occupations. The incentive to replace labour with AI-capital will be high. In 2017, McKinsey & Company estimated that across 800 occupations, nearly half of the activities for which people are collectively paid approximately US$ 16 trillion in wages in the global economy could potentially be automated using currently demonstrated technologies.^31^ This figure is likely an underestimate given the more recent advancements in generative AI. To remain nationally and globally competitive, companies across industries will seek to reduce costs, maintain or increase productivity, and remain relevant through the adoption of this transformative technology. Without intervention, there is a considerable risk of multifaceted disruptions that market forces alone cannot correct.

### Traditional responses

In response to a decline in consumer demand, traditional government and central bank interventions such as fiscal and monetary policies might be less effective as generative AI-induced job displacement is more than a temporary market disruption. For instance, temporary government stimulus or tax incentives may have limited impact if generative AI-induced job displacement causes individuals to save rather than spend, in anticipation of challenging times ahead. Similarly, monetary policy measures, such as reduced interest rates or quantitative easing, could be less effective if businesses are reluctant to invest due to concerns about longer-term demand suppression. These potential limitations underscore the need for early recognition and mitigation of risks, as well as the implementation of mechanisms to prevent a self-perpetuating cycle of recessionary pressures that could undermine economic stability, democracy and social well-being.

### Low and middle-income countries

There are additional implications of generative AI-related job displacement for low- and middle-income countries both in terms of their exposure to risks and capacity for governments to respond. The implications will vary based on factors such as national income, access to generative AI-capital and supporting infrastructure, economic structure, industry composition, regulatory capacity, social safety nets and their degree of integration with the global economy. While generative AI might initially be inaccessible or unaffordable in low-resource settings due to the cost of computing power and the data to train large language models that ensure privacy and data sovereignty, the technology is improving at a rapid pace. As the high costs of training AI models decrease, deploying this technology in low- and middle-income countries could become affordable, thus facilitating its spread. The prospect of widespread accessibility of generative AI offers substantial opportunities for low- and middle-income countries, particularly through existing mobile phone technology. Among these opportunities, generative AI could help support entrepreneurship and mitigate skills shortages across various areas including health care, education, software development, data analysis and decision-making, and enable climate-resilient infrastructure development and engineering, thereby driving overall development.^32^^,^^33^

However, there are also risks. The economies of many low- and middle-income countries depend on offshored work involving non-routine and routine cognitive tasks, such as information technology services, customer support and accounting.^34^ These jobs, previously considered safe from automation due to their cognitive nature, are now threatened by generative AI, which can perform these tasks at or above human-level performance. Traditionally, high-income countries have offshored these tasks to low- and middle-income countries to benefit from lower labour costs. However, it may soon become more economically viable to reshore these tasks. This scenario could lead to significant job losses in low- and middle-income countries. Additionally, economic instability in high-income countries due to generative AI-related job displacement could result in reduced foreign investment and aid to low- and middle-income countries, as well as decreased demand for their exports, negatively affecting their economies. 

Between- and within-country inequality could also be exacerbated. As generative AI-capital becomes a dominant factor of production, the profits are likely to concentrate among owners of AI-capital, predominantly in high-income countries or among the wealthy elites in low- and middle-income countries. Globally, the challenge lies in leveraging the potential benefits of generative AI while mitigating risks as countries transition to an age of intelligence.

## Call for a proactive response

While we are at an early stage of adoption of generative AI, the rapid advancement of this technology demands an immediate and proactive response. The nature of this response, however, depends on our collective vision for the future. Will we choose to uphold the status quo by prioritizing free-market principles?^35^ Or will we pursue a new direction where human contributions are harmoniously integrated with generative AI and valued beyond the capacity to generate profit? The latter aligns with the World Health Assembly resolution WHA77.13 *Economics of Health for All* passed in May 2024,^36^ which calls on Member States, international and regional financial institutions and other stakeholders, to reorient economic systems towards enhancing collective health and well-being. Furthermore, this vision resonates with the recent United Nations Pact for the Future, specifically its Global Digital Compact, which emphasizes the importance of regulating artificial intelligence and ensuring that AI advancements benefit society and future generations.^37^ The disruption caused by generative AI provides a unique impetus to achieve this reorientation by establishing a new social contract to prevent the potential harm of generative AI-driven job displacement. To realize this vision and avert the possible harms of generative AI, government responses should include a combination of fiscal policy, regulation, progressive social policies, integrated with health sector strategies and frameworks for data-knowledge ownership (Box 1).

Box 1 **A summary of possible proactive government responses^a^**

**A fiscal policy response**

The response should seek to ensure that the benefits of generative AI are distributed equitably across society. Example fiscal policy responses are:

- The entrepreneurial state^38^ strategy promotes that governments should retain a fair share of the benefits, such as equity, intellectual property rights or financial returns, from public sector investments in research and innovation. Government investments have contributed to the development of internet, GPS, touchscreen displays used in smart phones, life-saving pharmaceuticals and advancements in renewable energy technologies. However, these investments often yield no direct financial returns to the public sector. This strategy would generate revenue to reinvest in universal health care, social protections, education, and further research and development, creating a positive feedback loop.
- An automation tax^39^ could be used to disincentivize excessive labour displacement and address the negative externalities of increased automation. Integrated with adjustments to existing capital and labour taxes, the revenue could support displaced workers, finance vocational education and training initiatives, or contribute to social programmes.
- When redesigning tax frameworks, governments should balance disincentivizing excessive job displacement with preserving generative AI's potential to revitalize sluggish productivity growth and enhance work quality.^40^
- In countries with employment-based health insurance, governments should establish or extend universal coverage or public funding options to ensure health-care access remains unaffected by potential increases in unemployment.

The WHO Council for Economics of Health for All has argued that such fiscal policies are not just important for economic development, they also support population health by both limiting damage from economic displacement and ensuring a fairer, more sustainable distribution of productivity gains associated with technological change.^41^

**Regulation**

Effective regulation will require new evidence, analytic tools and frameworks to ensure the relevant knowledge guides changes in practice.

- Systems modelling and simulation (accounting for complex dynamics and feedback loops)^42^ should be used by governments to determine country-specific economy-wide AI-capital-to-labour ratio thresholds and assess sector-specific sensitivities. For example, in Australia, excessive job losses in the professional, scientific and technical services sector, which employs 9% of the workforce as of 2023, could shift the economy closer to the recessionary threshold than similar losses in the mining sector, which employs 2% of the workforce.^43^
- Governments could institute a regulatory framework to dynamically monitor labour displacement and maintain the AI-capital-to-labour ratio above the critical threshold.

**Progressive social policies**

A range of social policy initiatives can address the challenges of generative AI-driven job displacement. For example:

- A social production wage offers a living wage to those aged 16 years and older in exchange for engagement in socially productive activities.^44^^,^^45^ Unlike traditional welfare, this policy would strengthen community cohesion, promote inclusivity, reduce loneliness, and enhance the Mental Wealth of nations. This approach would reduce welfare stigma and promote a sense of productive purpose by engaging individuals in activities they are most suited to.
- Job guarantee policies ensure full employment by providing working-age individuals with adequately remunerated public service roles in community development initiatives, such as environmental projects and services for the elderly population.^46^ The job guarantee could enhance financial security and elevate the standard of living across society by effectively eliminating the threat of unemployment and associated health consequences.
- Reduced workdays with maintained salaries.^47^ For example, a four-day work week could translate generative AI-related productivity benefits into widespread improvements in work-life balance and mental health,^48^ thereby enhancing overall quality of life and societal well-being.
- To prevent contraction of the middle class, which is essential for a thriving economy, mechanisms to facilitate real wage growth may be necessary in labour-intensive sectors, particularly those in the foundational economy, such as health care, education, childcare and long-term care.
- Initiatives to support entrepreneurship, affordable housing and cost of living measures to improve the standard of living for workers in lower-paid roles or the unemployed may also be beneficial.

**Frameworks for data-knowledge ownership**

To ensure distributive justice, prevent exploitation and avoid exacerbating inequality, which affect health and well-being, governments could:

- Implement consent processes that enable individuals to consent to the use of their data and the methods by which they are processed, managed, analysed and used, ensuring transparency and control.
- Establish frameworks, such as data cooperatives or trusts,^49^^,^^50^ to recognize and provide fair remuneration for individuals’ contributions of data and cognitive processes that enrich generative AI systems.

**Integrated health sector response**

The health system can be strengthened with appropriate use of generative AI. For example through:

- AI-driven workforce augmentation and job creation. Governments can invest in AI customization to assist in diagnostics, treatment innovations, patient triage and administrative tasks, freeing health workers to focus on direct patient care. Governments can harness these AI-derived efficiencies to create new jobs and scale up preventive care programmes.
- Integrated AI-enhanced resilience hubs. Governments can establish integrated mental wealth hubs that provide job retraining, reskilling, mental health support and financial counselling, using AI to streamline services and improve access.
- Preventive health credit. Governments can implement a system of preventive health credits supported by generative AI, encouraging individuals to engage in proactive health management activities. Credits can be redeemed for health-care services or financial support, promoting both health and economic resilience.

The health system also plays an important role in mitigating the negative externalities associated with generative AI’s adoption across the economy. For example:

- Strengthening community health networks. The health sector can enhance community health networks to provide robust support systems, focusing on preventive care, mental health services, and social support, ensuring resilience against economic disruptions.
- Implementing collaborative health policy reforms. Structural health policy reforms can address systemic barriers to health-care access, such as updating reimbursement models, ensuring sustainable funding for public health programmes and fostering cross-sector collaboration to improve overall health system resilience.

AI: artificial intelligence; GPS: Global positioning system; WHO: World Health Organization. ^a^ Further recommendations for research and policy actions are provided elsewhere.^30^^,^^47^

## Conclusion

Generative AI, with its capacity to handle both routine and non-routine cognitive tasks, challenges conventional assumptions about job susceptibility to displacement. As this technology rapidly evolves, the belief that market forces will self-correct by creating sufficient new jobs appears overly optimistic. Instead, generative AI-driven productivity growth could paradoxically trigger recessionary pressures. These pressures depend on the complex interplay between the speed of generative AI adoption, the scale of job displacement versus new job creation, and the nature and effectiveness of policy responses. A realistic understanding of generative AI's potential to reshape the job market is crucial, as this technology could exacerbate social disparities, reducing social mobility and cohesion while heightening social tensions. This scenario underscores the urgent need for proactive policy responses and a new social contract inspired by an economics of health for all approach, to ensure a sustainable, inclusive and resilient economic and social future in this era of rapid technological advancement.
